# A Case Report of Uterine Body Constriction Precluding Normal Parturition Leading to Dystocia in a Mare

**DOI:** 10.3390/vetsci10020139

**Published:** 2023-02-10

**Authors:** Jaclyn Willette, Allison Gerras, Dodd Sledge, Drew Koch

**Affiliations:** 1 Department of Large Animal Clinical Sciences, College of Veterinary Medicine, Michigan State University, East Lansing, MI 48824, USA; 2 Department of Pathobiology and Diagnostic Investigation, College of Veterinary Medicine, Michigan State University, Lansing, MI 48824, USA; 3 Veterinary Diagnostic Laboratory, College of Veterinary Medicine, Michigan State University, Lansing, MI 48824, USA; 4 Department of Clinical Sciences, College of Veterinary Medicine, North Carolina State University, Raleigh, NC 27606, USA

**Keywords:** equine, dystocia, uterus, broad ligament, constriction

## Abstract

**Simple Summary:**

The presentation of equine dystocias to a referral clinic varies significantly. This case report describes the clinical and postmortem findings of a case of uterine body constriction in a late gestation mare that precluded normal vaginal delivery, ultimately resulting in fetal mortality with humane euthanasia of the mare. Gross examination revealed a circumferential, tan, fibrous band at the base of the uterine body that constricted the uterus and was adhered to the left and right ovaries, supporting the diagnosis of a uterine body constriction.

**Abstract:**

A 13-year-old multiparous Quarter Horse mare was presented to the Michigan State University’s, Large Animal Emergency service for dystocia. Clinical evaluation revealed a minimally dilated cervix on vaginal examination, with a palpable deceased fetus. Postmortem evaluation following owner-elected humane euthanasia revealed a circumferential, tan, fibrous band at the base of the uterine body that constricted the uterus and was adhered to the left and right ovaries. A routine histologic section of the incarcerating cord attached to the ovary consisted predominately of dense fibrous connective tissue, large blood vessels, and a central oviduct suggestive of a rent in the broad ligament. To the authors’ knowledge, this is the first case report to describe uterine body constriction that precluded vaginal delivery of a fetus in a late gestation mare.

## 1. Introduction

Equine dystocia is a true emergency requiring prompt medical and potentially surgical intervention to minimize the morbidity and mortality for both the mare and foal [1,2,3,4,5]. The time period between the onset of dystocia and its resolution is a significant determinant of outcome [5]. A retrospective analysis of a referral hospital population of equine dystocias concluded that each 10-minute increase in the duration of Stage II labor beyond 30 min was associated with a 10% increase in the risk of a foal being born deceased and a 16% increase in the risk of the foal not surviving to hospital discharge [5].

Fetal malposition within the birth canal is the most prevalent cause of dystocia in horses [1,6,7]. Resolution of dystocia involves a progression of procedures in a time-sensitive manner from assisted vaginal delivery (AVD) while the mare remains standing to controlled vaginal delivery (CVD) with the mare anesthetized in dorsal recumbency and, finally, to either fetotomy, cesarean section (alive or terminal), or humane euthanasia [5]. Additionally, in predominantly late gestation mares, an infrequent but serious cause of dystocia or signs of colic can be attributable to uterine torsion [8]. Techniques for the resolution of uterine torsion include manual rotation via a hand passed through the cervix, rolling under anesthesia, standing flank laparotomy, and ventral midline celiotomy. The goal of the aforementioned techniques is ultimately the resolution of the torsion to allow for a normal vaginal delivery [8]. Despite this range of known interventions, in the late gestation mare, these interventions may not lead to resolution in a gravid uterus [9]. Clearly, the resolution of equine dystocia requires immediate determination of the cause of the dystocia combined with appropriate intervention. 

This report describes an instance of uterine body constriction presenting to a referral institution as a suspected uterine torsion, resulting in dystocia and precluding vaginal delivery in a late gestation mare. To the authors’ knowledge, this is the first case report describing this as an etiology of dystocia in a multiparous mare. 

## 2. Case History

A 13-year-old multiparous Quarter Horse mare was presented to the Michigan State University’s Large, Animal Emergency service for dystocia. The mare was being managed at a reputable breeding and foaling facility and was at day 332 gestation according to her live-cover breeding date. The managers had noticed the mare had intermittent mild signs of discomfort, which mostly included laying down in the pasture. This was interpreted as potential Stage I labor, approximately twenty-four hours prior to presentation to the referral institution. Due to a lack of further progression into Stage II labor or worsening signs of discomfort throughout the day into the evening, further medical intervention was not elected at that time. The following morning, the mare was found in lateral recumbency and unwilling to rise. Evaluation by the referring veterinarian revealed the mare was tachycardic and tachypneic. A vaginal examination revealed no cervical dilation. Rectal examination was not definitive as the broad ligaments were unable to be traced due to significant straining by the mare. Referral was elected due to concerns about a potential uterine torsion. 

Previous medical history relevant to the mare’s clinical status included a mild to moderate colic episode when the mare was at approximately 4 months gestation. The colic episode was further described as an acute episode in which the mare suddenly went down and rolled in the pasture for ~10 min. The mare was administered a single-intravenous flunixin meglumine dose by the owners, and the colic episode resolved without further incident. No recurrence of colic was noted, and veterinary evaluation was not elected due to a lack of persistent clinical signs. This was the mare’s seventh foal with no previous history of dystocia.

## 3. Clinical Findings and Diagnosis

On presentation, the mare was anxious and displaying moderate colic signs. The mare was moderately tachycardic (60 beats/min) and had mild pyrexia (38.61 °C). Mucus membranes were hyperemic with a rapid capillary refill time of less than one second. Packed cell volume and total solids were 42% and 7.0 g/dL, respectively. A complete blood count revealed mild leukocytosis of 10,000 total white blood cells/uL (reference range: 4700–9500 cells/uL). Serum chemistry revealed mild hypernatremia of 143 mmol/L (reference range: 129–138 mmol/L), mild hyperchloremia of 103 mmol/L (reference range: 93–101 mmol/L), mild hyperalbuminemia of 4.0 g/dL (reference range: 2.8–3.8 g/dL), moderate hyperglycemia of 138 mg/dL (reference range: 77–105 mg/dL), mildly elevated gamma-glutamyl transferase (GGT) of 23 U/L (reference range: 8–20 U/L), and a mild elevation in total bilirubin of 2.1 mg/dL (reference range: 0.6–1.9 mg/dL) further characterized by a mildly elevated indirect bilirubin of 1.7 mg/dL (reference range: 0.5–1.5 mg/dL) and normal direct bilirubin. Venous blood gas revealed a normal pH of 7.42 with a moderate decrease in bicarbonate at 18.1 mmol/L (reference range: 26.9–35.5 mmol/L). Additionally, the venous blood gas was consistent with the serum chemistry findings concluding mild hypernatremia of 143 mmol/L (reference range: 132–140 mmol/L), hypocalcemia of 5.8 mg/dL (reference range: 6.1–7.1 mg/dL), mild hypomagnesemia of 0.9 mg/dL (reference range: 1.0–1.3 mg/dL), and mild hyperlactatemia of 1.8 mmol/L (reference range: 0.3–1.1 mmol/L) (the abnormal hematologic parameters are summarized in Table 1). The remainder of the hematologic findings were within normal limits.

Abdominal palpation per rectum revealed palpable broad ligaments suspending the uterus in the caudal abdomen, with no apparent tension in the ligaments between the uterus and pelvic origin, suggesting uterine torsion was unlikely. A moderately firm uterine body was palpable with no evidence of fetal movement upon manipulation per rectum. Both transcutaneous abdominal and transrectal ultrasonography revealed no evidence of a fetal heartbeat. In addition, there was no subjective increased free peritoneal fluid or other abnormalities noted on full abdominal ultrasonographic evaluation.

Vaginal examination revealed a partially dilated cervix (approximately 4 cm) with mild amounts of serosanguineous to purulent malodorous exudate. Digital examination of the fetus through the external cervical os revealed no fetal movement consistent with foal death. The foal was in a transverse, head-back position. Due to the inability to improve cervical dilation and malpresentation of the fetus, controlled vaginal delivery was not a viable option, and a cesarean section was recommended. The owners elected humane euthanasia at that time due to financial constraints and orthopedic co-morbidities.

## 4. Case Outcome 

**Figure 1:**
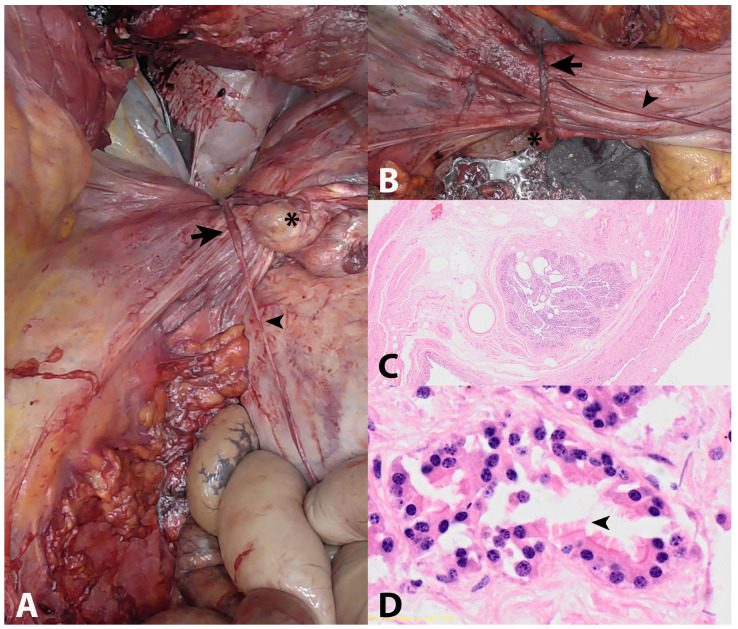
Gross and microscopic images of the uterine body constriction: (**A**,**B**) Gross images of uterus circumferentially bound by thick fibrous tissue (arrow) attached to the right ovary (star) and fatty stalk (arrowhead); (**C**) Low-magnification microscopic image of the fibrous band containing oviduct; (**D**) Higher-magnification microscopic image of oviduct containing ciliated epithelium (arrow).

A full postmortem examination with pathologic-anatomical inspection of a broad spectrum of different organs with histologic tissue evaluation was performed. Significant findings from gross anatomical inspection revealed a circumferential, tan, fibrous band at the base of the uterine body that constricted the uterus and was adhered to the left and right ovaries. On histologic examination, the representative section of the band was composed of the oviduct, fibrous connective tissue, and large blood vessels. This band of tissue did not connect to the body wall. The peritoneal surface of the adjacent uterine tissue was light pink, and there was no evidence of hemorrhage. Additionally, attached to this band of tissue was an approximately 1 m long, tan, light yellow to pink, and slightly firm to friable band of tissue consistent with a fatty stalk that extended into the cranial mesenteric fat and was easily removed with traction (Figure 1A,B). There was no overt evidence of a lipoma at the termination of this stalk or further evidence of adhesions within the abdomen. The right uterine horn contained a female fetus that had a crown to rump length of 92 cm. There were no other significant gross lesions.

Routinely, hematoxylin- and eosin-stained representative sections of the trachea, heart, lung, liver, kidney, spleen, stomach, uterus, placenta, ovary, uterine band, jejunum, ileum, and colon were examined histologically. The ovarian tissue demonstrated decreased cellularity and was predominately composed of bundles of spindle ovarian stromal cells, large blood vessels, and a tertiary ovarian follicle with a markedly thickened and hyalinized basement membrane. The ovarian surface was lined by low cuboidal epithelium. The incarcerating cord was predominately composed of dense fibrous connective tissue, large blood vessels, and a central oviduct (Figure 1C,D). Histologic sections of the placenta adjacent to the constrictive band revealed superficial papillary projections with superficial small, round, basophilic mineralized foci. There were no significant abnormalities within sections of the uterus nor evidence of vascular compromise. There were no other significant microscopic lesions. Histopathologic conclusions were that the tissue that was constricting the uterine body was composed of an oviduct and dense fibrous connective tissue that was adhered to two ovaries and a second pedunculated band of elongated suspected mesenteric fat.

## 5. Discussion

Fetal malposition within the birth canal is the most prevalent cause of dystocia in horses [1,6,7]. However, the present report describes another rare complication of parturition that would preclude normal vaginal delivery, requiring cesarean section for resolution. The equine uterus is attached to the abdominal and pelvic walls by two extensive peritoneal folds deemed the broad ligaments or mesometrium [10]. Each broad ligament contains marked vasculature, nerves, lymphatics, fat, connective tissue, and smooth muscle. The uterine blood supply, which courses through the broad ligament, originates from the uterine branch of the vaginal artery, the uterine artery, and the uterine branch of the ovarian artery [11]. 

The exact pathogenesis for this lesion is unclear, but a proposed mechanism is the rupture of the broad ligament and mesenteric fat during early pregnancy with secondary stretching of the ligaments and oviduct throughout gestation. Potentially, the gravid uterus then grew through this rent. The tissue constricting the uterine body, as concluded via histologic evaluation, was composed of the oviduct and dense fibrous connective tissue that was adhered to two degenerate ovaries and a second pedunculated band of elongated fat that extended into the mesentery. Only a representative section of the constricting band was examined histologically; as such, the presence of the oviduct throughout the entire length cannot be definitively confirmed but is suspected. Regardless, this band likely represents remnant mesometerium, given the presence of the oviduct in the histologic section examined led to constriction. There was no evidence of uterine torsion or vascular compromise within the uterus both on gross evaluation and histologically. 

At the time of necropsy, there was minimal to absent mesometrial tissue upon gross and microscopic examination. The proposed mechanism of a rent in the mesometrium is possible detachment, stretching, or stripping of the tissue as the uterus enlarged, leading to constriction at the time of parturition. In human medicine, there are various classifications of broad ligament herniations that generally cause incarceration of intestinal loops, and surgical correction is required [12,13]. This has also been reported in mares [14,15]. In this case, there was no evidence of intestinal entrapment, although the origin of the band of stroma and fat extending from the band and entrapping the uterus and the mesentery is unclear. Other possibilities to consider for the origin of the mesenteric band from the abdomen may reflect a mesenteric adhesion, a free portion of the ruptured broad ligament, or less likely the remnant of a pedunculated lipoma, although no lipoma was found. Based on the entire examination, the most likely differential was an adhered free portion of a ruptured broad ligament as the origin for this band.

Reports of a broad ligament tearing during labor have occurred as a rare complication of parturition in human medicine. A case report by Soleymanimajd et al. (2007) described a longitudinal full-thickness tear on the posterior leaf of the left broad ligament of a female that precluded normal vaginal delivery. This tear disrupted the adjacent venous sinuses, leading to hemoperitoneum [16]. The incidence of broad ligament injury is rare and is mostly associated with abdominal or pelvic trauma or occurs post-delivery in human medicine [17]. Suggested pathophysiologic factors contributing to spontaneous rupture of the ligament and uterine vessels in human medicine include arteriovenous malformation, increased intravenous pressure, free anastomosis of the utero-ovarian vessels in the broad ligament, absence of valves of the ovarian veins, and weakness of vessel walls [18]. Prior to presentation at our hospital, the mare suffered an acute colic episode that resolved without further medical intervention. It is plausible that this episode of discomfort was due to broad ligament tearing or broad ligament rupture leading later to constriction as the gravid horn of the uterus enlarged throughout parturition through this rent in the broad ligament. 

Interestingly, the mare was not reported to have any other colic episodes or signs of discomfort for the duration of her pregnancy until her dystocia. Pregnant mares at any stage of gestation with signs of abdominal discomfort (colic) can present with varying degrees of pain and clinical signs, creating a diagnostic and therapeutic challenge [19,20,21,22,23]. Potentially, the mare exhibited mild intermittent signs of discomfort that resolved without medical intervention. Explanations for why these signs, if present, were missed throughout her pregnancy were that the mare was housed predominantly on pasture with several other horses. Therefore, mild generalized signs of colic, including decreased manure production, hyporexia, and flank watching, could have been missed during the remainder of her pregnancy [19,20,21,22,23]. 

Periparturient, preparturient, and postpartum hemorrhage and associated broad ligament hematomas secondary to arterial injury are an often-reported complication of equine parturition [24,25,26,27,28,29,30]. Arterial rupture has been shown to be age-related, with the probability increasing with each pregnancy beyond 10 years of age [27,28,30,31,32]. As previously reported by Ueno et al. 2010, arterial injuries that led to broad ligament hematoma in peripartum mares occurred most frequently in the proximal uterine artery, and atrophy of smooth muscle cells with fibrosis of the arterial wall was one of the predisposing factors in aged and multiparous mares [29]. Although no arterial compromise or hematomas were noted via the clinical diagnostics and histologically in the present case, a plausible pathophysiology preceding ligament rupture could be the atrophy of the connective tissue, smooth muscle, and nerves contributing to a weakened broad ligament. These potential changes, combined with the mare’s age and multiparous history, could have weakened the ligament and subsequently led to the ligament’s rupture, manifesting as the acute transient colic episode noted in early gestation [27,28,29,30,31,32]. Subsequently, this would have led the gravid horn to become continuously more constricted as the pregnancy progressed, preventing normal dilation of the cervix and normal progression of labor. 

Uterine torsion is a rare complication in late gestation mares that often leads to the exhibition of colic signs [8]. In horses, vaginal examination is usually not diagnostic for uterine torsion since the torsion is entirely cranial to the cervix. Instead, a presumptive diagnosis is determined through abdominal palpation per rectum, where a taut broad ligament coursing dorsal to the caudal aspect of the uterus in the direction of the torsion can be identified [11]. In the present case, there were no palpable taut broad ligaments coursing in an abnormal direction, leading clinicians to be less suspicious of uterine torsion. However, the most overt antemortem finding on clinical examination that prompted the recommendation of cesarean section was the inadequate cervical dilation that precluded controlled vaginal delivery. Retrospectively, the attending clinicians were potentially unable to palpate cranially to the constricted uterine body. Accordingly, this constriction could have also prevented normal dilation of the cervix and precluded any vaginal delivery. The combination of antemortem and postmortem findings, in this case, lead the authors to conclude that when there is clinical evidence of uterine body constriction, surgical intervention via a ventral midline cesarean section remains the primary recommendation for the management of these cases.

## 6. Conclusions

This case report describes a uterine body constriction precluding vaginal delivery in a late gestation mare that was presented to a referral institution for dystocia. The proposed mechanism of constriction was a rupture of the broad ligament, leading to a rent entrapping the gravid uterine horn as the fetus matured throughout gestation. Clinicians should consider uterine body constriction as a differential diagnosis in mares presenting for dystocia with a partially dilated cervix with inadequate progression of labor.

## Figures and Tables

**Table 1:** A summary of the abnormal hematologic values measured on initial evaluation of both the serum chemistry and venous blood gas.

Summary of Abnormal Hematologic Values		
	Serum Chemistry	
Measurement	Result	Reference Range&Units
Sodium Chloride Albumin Glucose GGT Total bilirubin Indirect bilirubin	143 103 4 138 23 2.1 1.7	129–138 mmol/L 93–101 mmol/L 2.8–3.8 g/dL 77–105 mg/dL 8–20 U/L 0.6–1.9 mg/dL 0.5–1.5 mg/dL
Venous Blood Gas		
Measurement	Result	Reference Range&Units
pH Bicarbonate Sodium Calcium Magnesium Lactate	7.42 18.1 143 5.8 0.9 1.8	7.40–7.45 26.9–35.5 mmol/L 132–140 mmol/L 6.1–7.1 mg/dL 1.0–1.3 mg/dL 0.3–1.1 mmol/L

## Data Availability

Not applicable.

## References

[1] Roberts S.J. (1986). Veterinary Obstetrics and Genital Diseases (Theriogenology 3rd edition).

[2] Byron C.R., Embertson R.M., Bernard W.V., Hance S.R., Bramlage L.R., Hopper S.A. (2002). Dystocia in a referral hospital setting: Approach and results. Equine Vet. J..

[3] Embertson R.M., McKinnon A.O., Squires E.O., Vaala W.E. (2011). Referral Dystocias, Equine Reproduction.

[4] Lu K.G., Barr B.S., Embertson R.M., Dallap B.L. (2006). Dystocia—A true emergency. Clin. Tech. Equine Pract..

[5] Norton J.L., Dallap B.L., Johnston J.K., Palmer J.E., Sertich P.L., Boston R., Wilkins P. (2007). Retrospective study of dystocia in mares at a referral hospital. Equine Vet. J..

[6] Frazer G.S., Perkins N.R., Blanchard T.L., Orsini J., Threlfall W.R. (1997). Prevalence of fetal maldispositions in equine referral hospital dystocias. Equine Vet. J..

[7] Rossdale P.D., Ricketts S.W. (1974). The Practice of Equine Stud Medicine.

[8] Martens K.A., Govaere J.L., Hoogewijs M.K., Lefevre L., Nollet H., Vlaminck L., Chiers K., de Kruif A. (2008). Uterine torsion in the mare: A review and three case reports. Vlaams Diergeneeskd Tijdschr..

[9] Lopez C., Carmona J.U. (2010). Uterine torsion diagnosed in a mare at 515 days’ gestation. Equine Vet. Educ..

[10] Getty R. (1975). The Anatomy of the Domestic Animals.

[11] Auer J.A., Stick J.A., Kümmerle J.M., Prange T. (2019). Equine Surgery.

[12] Hashimoto Y., Kanda T., Chida T., Suda K. (2020). Recurrence hernia in the broad ligament of the uterus: A case report. Surg. Case Rep..

[13] Cilley R.E., Poterack K., Lemmer J.H., Dafoe D.C. (1986). Defects of the broad ligament of the uterus. Am. J. Gastroenterol..

[14] Steward S.K., Bauck A.G., Zoll W., Conway J.A., Freeman D.E. (2018). Small intestinal strangulation in a tear in the mesometrium of a nonpregnant mare. Equine Vet. Educ..

[15] Crecan C., Morar I., Mircean M.V., Oros D., Muresan A., Taulescu M. (2019). Small intestinal herniation through the broad ligament in a mare outside of the gestation period–a case report. Acta Vet. Brno.

[16] Soleymanimajd H., Datta S., Iqbal R. (2007). Spontaneous broad ligament tear in early labour. J. Obstet. Gynaecol..

[17] Chowdhury R.R., Ahern T., McKenzie-Gray B. (2004). Prelabour rupture of the broad ligament in a primigravida. Brit. J. Obstet. Gynecol..

[18] Moreira A., Reynolds A., Baptista P., Costa A.R., Bernardes J. (2009). Case report: Intra-partum utero-ovarian vessels rupture. Arch Gynecol. Obstet..

[19] Steel C.M. (2001). Gibson, KT. Colic in the pregnant and periparturient mare. Equine Vet. Educ..

[20] Santschi E.M., Slone D.E., Gronwall R., Juzwiak J.S., Moll H.D. (1991). Types of colic and frequency of post colic abortion in pregnant mares: 105 cases (1984–1988). J. Am. Vet. Med. Assoc..

[21] Boening K.J., Leendertse I.P. (1993). Review of 115 cases of colic in the pregnant mare. Equine Vet. J..

[22] Chenier T.S., Whitehead A.E. (2009). Foaling rates and risk factors for abortion in pregnant mares presented for medical or surgical treatment of colic: 153 cases (1993–2005). Can. Ve.t J..

[23] Perkins N.R., Frazer G.S. (1994). Reproductive emergencies in the mare. Vet. Clin. North Am. Equine Pract..

[24] Britt B.L., Robinson N.E. (2003). Postpartum hemorrhage. Current Therapy in Equine Medicine 5.

[25] Frazer G.S., Reed S.M., Bayly W.M., Sellon D.C. (2004). Periparturient hemorrhage. Equine Internal Medicine.

[26] Lofstedt R. (1994). Haemorrhage associated with pregnancy and parturition. Equine Vet. Educ..

[27] LeBlanc M.M., Colahan P.T., Mayhew I.G., Merritt A.M., Moore J.N. (1991). Diseases of the reproductive system: The mare. Equine Medicine and Surgery.

[28] Youngquist R.S., Threlfall W.R., Morrow D.A. (1997). Current Therapy in Theriogenology 2.

[29] Ueno T., Nambo Y., Tajima Y., Umemura T. (2010). Pathology of lethal peripartum broad ligament haematoma in 31 Thoroughbred mares. Equine Vet. J..

[30] Oikawa M.A., Nambo Y., Miyamoto M., Miura H., Kikuchi M. (2009). Ohnami, Y. Postpartum Massive Hematoma within the Broad Ligament of the Uterus in a Broodmare Possibly Caused by Rupture of the Uterine Artery. J. Equine Sci..

[31] Rooney J.R., Robertson J.L., Rooney J.R., Robertson J.L. (1996). Rupture of the uterine, ovarian, iliac arteries. Equine Pathology.

[32] Williams N. (2001). Uterine artery rupture. Equine Dis. Q..

